# Supplementary material for: Phantom vortices: hidden angular momentum in ultracold dilute Bose-Einstein condensates
Source: Sci Rep. 2017 Jan 16;7:40122. doi: 10.1038/srep40122 (PMC5238373; doi:10.1038/srep40122)
Supplement: Supplementary Material [file srep40122-s4.pdf]

Phantom vortices: hidden angular momentum in ultracold dilute Bose-Einstein condensates

Supplementary Material

Storm E. Weiner*

Department of Chemistry, University of California at Berkeley, CA, USA

Marios C. Tsatsos

Instituto de Física de São Carlos, Universidade de São Paulo, São Carlos, São Paulo, Brazil

Lorenz S. Cederbaum

Theoretische Chemie, Physikalisch–Chemisches Institut,

Universität Heidelberg, Heidelberg, Germany

Axel U. J. Lode

Department of Physics, University of Basel, Basel, Switzerland

(Dated: December 19, 2016)

* Corresponding author: StormWeiner@Berkeley.edu

In this supplementary document, we define the MCTDHB method we use to solve the time-dependent many-body Schrödinger equation and specify the numerical details of our computations. Furthermore, we give a detailed description of the quantities of interest analyzed in the main text, and an analytical discussion of the existence of fragmented vortices. We then compare our results to a GP mean-field description and assess the generality of our findings for 10 to 10^4 particles. Finally, we provide captions for the enclosed videos and compare our results for short-ranged interactions to computations with a zero ranged Dirac-delta pseudopotential.

MCTDHB Method and Numerical Details

We solve the time-dependent Schrödinger equation with the Hamiltonian described in the main text using the MCTDHB method [1]. MCTDHB uses the ansatz,

$$|\Psi\rangle = \sum_{\{\mathbf{n}\}} C_{\mathbf{n}}(t) |\mathbf{n}; t\rangle \tag{1}$$

where $|\mathbf{n}; t\rangle = |n_1, ..., n_M; t\rangle$ is a permanent of M orthonormal orbitals which *explicitly depend on time*, i.e. a fully symmetrized product of at most M distinct single particle states. Using a time-dependent variational basis ensures the highest possible accuracy for a given M at all times [2]. In fact, each coefficient and each orbital are treated as independent variational parameters. The basis of the considered Hilbert space is hence a set of $\frac{(M+N-1)!}{N!(M-1)!}$ permanents. In the limit $M \to \infty$, the configurations $|\mathbf{n}; t\rangle$ span the complete $N-$particle Hilbert space and the expansion in Eq. 1 becomes formally exact. In practice, a small number of time-dependent single-particle states is enough to obtain numerically exact results [3, 4]. In the case of $M = 1$, Eq. 1 is the GP ansatz and the coupled system of nonlinear integro-differential equations of motion of MCTDHB also simplify to the time-dependent GP equation [1].

We use the recursive implementation of the MCTDHB theory [1] in the MCTDH-X package [5] for the simulations presented in this work. Computations using MCTDH-X employ a discrete variable representation (DVR) to represent the time-dependent orbitals and the one-body Hamiltonian operator as an expansion of a time-independent basis set, see for instance [6]. For the present work, plane waves were used as the primitive basis.

To check convergence of our computations with respect to the DVR we computed the energy of the ground state of $N = 100$ bosons in a parabolic trap while varying the number of DVR points used to represent the simulated domain of extension $[-8, 8] \times [-8, 8]$ at different widths of the short-range gaussian potential, $\sigma \in [0.1, 0.35]$. We varied the number of DVR points from 64×64 to 512×512. We found that the energy was converged up to the 16 significant digits with respect to the DVR using $\sigma = 0.25$ and 128×128 grid points. This choice of σ is well in the range of widths found to match the physics of contact interaction potentials in Ref. [7].

Furthermore, we repeated our simulation for the time-dependent potential of Eq. (5) of the manuscript, on a 256×256 grid. The resulting system – densities and observables – is practically identical to the 128×128 one. The total energy found at the end of the rotation protocol ($t = 499.0$) differs only by at most 0.29% while the angular momentum and largest natural occupation differ by less than 0.03%. See Fig. 2 for a comparison of the angular momentum, first natural occupation, and energy for short- and zero-ranged interactions on the different mentioned grids.

The bulk of our simulations considers $N = 100$ and $M = 4$, which yields $N_C = 176\ 851$ configurations. The integration of the MCTDHB equations of motion comprises the solution of two coupled sets of generally non-linear integro-differential equations of motion, see [1, 4] for details. The set of coefficient equations is linear and of dimension N_C and the set of orbital equations is non-linear, integro-differential and of dimension $M \times 128 \times 128$. It is important to note that the number of configurations in the mean-field case, $M = 1$, is $N_C = 1$. Since the configurations are mutually orthonormal functions, the number of coefficients directly measures the size of the Hilbert space in comparison to the mean-field case. In dimensionful units [8], the simulation time $t = 500.0$ corresponds to 2.42 seconds and the domain $[-8, 8] \times [-8, 8]$ corresponds to 12×12 micrometers.

Quantities of Interest

Here we provide the quantities which were analyzed throughout the main text, namely the density, one-body reduced density, natural occupations, natural orbitals, and 1st-order correlation function. The density, $\rho(\mathbf{r}; t)$, is given as the diagonal of the one-body reduced

density matrix:

$$\rho(\mathbf{r};t) = \rho^{(1)}(\mathbf{r}|\mathbf{r};t) \tag{2}$$

The one-body reduced density matrix, $\rho^{(1)}$, for an N-body system is defined as the partial trace of the N-body density [9]:

$$\rho^{(1)}(\mathbf{r}_1|\mathbf{r}'_1;t) = N \int \Psi(\mathbf{r}_1, \mathbf{r}_2, ..., \mathbf{r}_N; t)\Psi^*(\mathbf{r}'_1, \mathbf{r}_2, ..., \mathbf{r}_N; t) d\mathbf{r}_2...\mathbf{r}_N, \tag{3}$$

where the Ψ is assumed to be normalized to 1. The one-body density matrix can be expanded in a complete basis $\{\xi_j(\mathbf{r};t)\}$,

$$\begin{aligned}\rho^{(1)}(\mathbf{r}|\mathbf{r}';t) &= \sum_{j,k} \rho_{jk}(t)\xi_j^*(\mathbf{r};t)\xi_k(\mathbf{r}';t) \\ &= \sum_j \rho_j^{(NO)}(t)\phi_j(\mathbf{r};t)\phi_j(\mathbf{r}';t),\end{aligned}$$

where we have diagonalized the matrix elements ρ_{kj} in the last line to obtain the eigenvalues $\rho_j^{(NO)}(t)$ and eigenfunctions $\{\phi_j(\mathbf{r};t)\}$, which are called the natural occupations and natural orbitals, respectively [1]. Since the trace operation in Eq. 3 is not one-to-one, there are numerous many-body wavefunctions with the same RDM. In fact, if all of the natural occupations are commensurate with the particle number, i.e. $n_i \equiv N\rho_i^{(NO)} \in \mathbb{N}$, then there is a mean-field wavefunction with the same RDM, namely $|\Psi_{MF};t\rangle = |n_1, ..., n_m; t\rangle$. The 1st-order correlation function, $g^{(1)}$, is defined in terms of the one-body density matrix [10]:

$$g^{(1)}(\mathbf{r}|\mathbf{r}';t) = \frac{\rho^{(1)}(\mathbf{r}|\mathbf{r}';t)}{\sqrt{\rho^{(1)}(\mathbf{r}|\mathbf{r};t)\rho^{(1)}(\mathbf{r}'|\mathbf{r}';t)}}. \tag{4}$$

The off-diagonal, $\mathbf{r} \neq \mathbf{r}'$, terms in $g^{(1)}$ and the orbitals ϕ_i are complex-valued. Thus it is instructive to plot their magnitudes and phases separately. We hence define the phase of $g^{(1)}(\mathbf{r}|\mathbf{r}';t)$ to be

$$S_g(\mathbf{r}|\mathbf{r}';t) \equiv \arg\left[g^{(1)}(\mathbf{r}|\mathbf{r}';t)\right], \tag{5}$$

and the orbital phases to be

$$S_i(\mathbf{r};t) = \arg\left[\phi_i(\mathbf{r};t)\right]. \tag{6}$$

For the sake of completeness, we likewise define the phase of the many-body wavefunction, Ψ, as

$$S_{MB}(\mathbf{r}_1, ..., \mathbf{r}_N; t) = \arg\left[\Psi(\mathbf{r}_1, ..., \mathbf{r}_N; t)\right]. \tag{7}$$

We choose to plot all phases on the interval $[-\pi, \pi]$, but any interval of the same length is equally valid. Furthermore, the global phase transformation,

$$\phi_i(\mathbf{r};t) \to e^{i\theta_i(t)}\phi_i(\mathbf{r};t)$$
$$C_\mathbf{n} \to e^{-i(\gamma + \sum_{i=1}^M \theta_i(t) n_i)} C_\mathbf{n} \qquad \forall \gamma, \theta_i \in \mathbb{R}$$

leaves the many-body wavefunction (Eq. 1) invariant. Phase shifting an orbital ϕ_i by θ_i is equivalent to plotting it in the interval $[-\pi + \theta_i, \pi + \theta_i]$ with all structures unchanged. Whereas, $g^{(1)}(\mathbf{r}|\mathbf{r}') \propto \rho^{(1)}(\mathbf{r}|\mathbf{r}') = \sum_{i=1}^M \rho_i^{(NO)} |\phi_i(\mathbf{r})||\phi_i(\mathbf{r}')| e^{i(S_i(\mathbf{r}) - S_i(\mathbf{r}'))}$ depends only on phase differences within the same orbital, and is thus invariant to global phase shifts. Therefore, the topological defects we see in the orbitals (Figs. 2 and 3 in the main text, Videos S1 and S2) and $g^{(1)}$ (Fig. 4 and Video S3) are unaffected by global phase transformations.

Analytical Discussion

In the main text we consider the possibility that a fragmented vortex exists in the total density. The simplest way this can happen is through the presence of one phantom vortex in each orbital with all cores coinciding, say at the origin, at some fixed time. This can be formally expressed in polar coordinates as

$$\phi_j(\mathbf{r}) = A_j(r) e^{iq_j\theta}, \tag{8}$$

where $A_j(r) \in \mathbb{R}$ is the radial amplitude and $q_j \in \mathbb{Z}$ is the vortex charge of the jth orbital. Furthermore, the constraint of a single vortex in each orbital requires that $A_j(0) = 0$, $A_j(r \to \infty) = 0$ and $A_j(r \neq 0) \neq 0$. Thus, either $A_j(r) \leq 0$ or $A_j(r) \geq 0$ for all r. Orthonormality of the orbitals then requires

$$\int_0^\infty A_j(r) A_k(r) r dr \int_0^{2\pi} e^{i(q_k - q_j)\theta} d\theta = 0, \quad \forall j \neq k. \tag{9}$$

The radial integral is strictly nonzero since the radial amplitudes cannot change sign. Thus, the angular integral must vanish. Using the orthonormality of Fourier modes, we conclude that $q_k \neq q_j$, i.e. the different phantom vortices must have different charges. This case is partially exemplified in ϕ_1 and ϕ_4 (Fig. 3b,e in the main text), which contain a charge 1 and 3 phantom vortex at the origin, respectively. No density vortex is observed because there are no phantom vortices at the origin in ϕ_2 or ϕ_3. There are many ghost vortices at the edge

of the orbitals which do not affect the present argument because they occur in areas where A_j is very small. If all the particles were to occupy only the fragments, ϕ_1 and ϕ_4, there would be a density vortex at the origin.

The other possibility to observe a fragmented vortex in the density is to have coincident phantom vortices of the *same* charge as well as noncoincident phantom vortices in regions where A_j is large enough to ensure orthonormality. For example, ϕ_2 and ϕ_3 (Fig. 2c,d in the main text) each have a charge 1 phantom vortex at the origin and several noncoincident charge 1 phantom vortices near the origin. If all the particles were to occupy only these two orbitals, there would be a density vortex at the origin.

In either case, the existence of a fragmented vortex visible in the total density requires *more than unit angular momentum* per particle. This directly contradicts the mean-field prediction. Our findings indicate that the many-body system tends to absorb more angular momentum from a perturbation than the mean-field system (see next paragraph).

Different Particle Numbers, Trap Anisotropies, Rotation Frequencies and Interparticle Interactions and Comparison to Mean-Field

Phantom vortices were found in various systems where the particle number N, trap anisotropy $\eta_{\max}$, rotation frequency ω and interparticle interaction strength λ_0 were varied as shown in Table 1.

Parameter	Range
N	10 to 10 000
λ_0	0.0017 to 1.9
η_{max}	0.05 to 0.40
ω	0.72 to 0.94

TABLE I. System parameters for the emergence of phantom vortices.

In order to estimate the generality of the emergence of fragmentation, we would like to discuss a scan through different particle numbers in detail. We keep all other dynamical parameters fixed, with $\omega = 0.78$ and $\eta_{max} = 0.10$, and vary the particle number from $N = 10$ to $N = 10\ 000$ with $M = 2$ orbitals. The interaction strength $g = \lambda_0(N-1) = 17.1$ is kept fixed, as it is the single relevant parameter in GP dynamics. We emphasize that GP theory

would predict exactly the same dynamics as N is varied with fixed g. Two orbitals are used because this is the minimum required to capture fragmentation. Should fragmentation not occur, the dynamics would be identical for all $M \geq 2$, and exactly mimic GP theory. We studied systems with up to $N = 10^4$ and found that fragmentation occurs on relevant time scales (Fig. 1). Importantly, after the trap becomes symmetric, all these calculations show a phantom vortex at the origin in the first orbital, and ghost vortices in both orbitals. Only ghost vortices are seen in the density. At the end of the rotating procedure, the stable angular momentum per particle ranged from 0.88 to 1.55. This further corroborates the claim that the time-dependent GP approximation systematically underestimates the quantity of angular momentum absorbed from the perturbation. It is important to stress that the phenomenon of phantom vortices exists over a large range of particle numbers also relevant to current experiments.

Comparison to Gross-Pitaevskii

We ran a calculation with $M = 1$ orbital, with the same one-body and two-body potential, to compare our results directly to the time-dependent GP theory. This check is necessary because the literature does not display the results of subcritical rotations in our parameter regime in detail [11, 12]. Furthermore, these treatments used a contact (delta) interaction while we use a Gaussian. We found that the GP system does not nucleate vortices in the density, but does show ghost vortices along the edge of the cloud. This agrees with our many-body treatment. However, the GP treatment results in a much smaller angular momentum $L_z/N = 0.46$ as compared to our computations which yielded $L_z/N = 1.25$ for $N = 100$ and $M = 4$. The many-body system absorbs significantly more angular momentum from an external perturbation.

Comparison to contact interaction

In the main manuscript we presented calculations with a short-range interaction, modeled by a Gaussian function. It is natural to ask whether the correlations and the appearance of phantom vortices have been triggered by the finite-range interparticle interaction. The answer is negative. A comparison to a simulation of an identical system but with a con-

tact interaction (Dirac-delta pseudopotential rather than a Gaussian) shows that the main characteristics of the system are unchanged and phantom vortices do form in a very similar configuration (Fig. 3). The initial total energies are close to identical in the two cases, whereas the contact-potential system accumulates slightly more energy and angular momentum at the end of the process (Fig. 2). The largest natural occupation number is around 65%, which is higher than its Gaussian counterpart. This shows that the contact interaction system exhibits a smaller fragmentation. The orbital densities are found to have a very similar structure to those in the main text with a Gaussian interaction: the first orbital shows a charge-1 vortex, the second two charge-1 vortices, the third is vortex-free and the last orbital features a large charge-3 vortex. However, since the first occupation is higher, the first phantom vortex is visible as a dip in the total density.

Movies

a. Video S1: `http://youtu.be/ezbdLWvSbBI`

Time evolution of the density, natural orbitals, and S_g: Movie corresponding to Figs. 2 and 3 of the main text. (a) shows the density $\rho(\mathbf{r};t)$, (b)-(e) and (g)-(j) show the squared magnitudes and phases of the natural orbitals, respectively, and (f) shows the phase of the one-body correlation function, $S_g(\mathbf{r}|0;t)$. After the onset of fragmentation at $t = 80$, the individual orbitals show a complicated vortex structure while the density is largely featureless. $S_g(\mathbf{r}|0;t)$ shows phase discontinuities characteristic of vortices even when no vortices are present in ρ.

b. Video S2: `http://youtu.be/whRL8haF4RA`

Scan of reference point in $g^{(1)}$ at $t = 115$: Movie corresponding to Fig. 4 of the main text. At a fixed time, $t = 115$, we plot $g^{(1)}(\mathbf{r}|\mathbf{r}')$ as $\mathbf{r}'$ is swept from -6 to 6 along the x-axis (panels a,c) and y-axis (panels b,d). We plot the magnitude in panels a,b and the phase, S_g, in panels c,d. There are topological defects that are associated with phantom vortices at each value of $\mathbf{r}'$.

c. Video S3: `http://youtu.be/gG7dprvRWGg`

Scan of reference point in $g^{(1)}$ at $t = 450$: Movie corresponding to Fig. 4 of the main text. At a fixed time, $t = 450$, we plot $g^{(1)}(\mathbf{r}|\mathbf{r}')$ as $\mathbf{r}'$ is swept from -6 to 6 along the x-axis (panels a,c) and y-axis (panels b,d). We plot the magnitude in panels a,b and the phase, S_g, in panels c,d. There are topological defects that are associated with phantom vortices at each value of $\mathbf{r}'$.

[1] Alon, O.E., Streltsov, A.I. & Cederbaum, L.S., Multiconfigurational time-dependent Hartree method for bosons: Many-body dynamics of bosonic systems. *Phys. Rev. A* **77**, 033613 (2008).

[2] Kramer, P. and Saraceno, M., Geometry of the Time-Dependent Variational Principle in Quantum Mechanics (Springer, 1981).

[3] Lode, A.U.J., Sakmann, K., Alon, O.E., Cederbaum, L.S. & Streltsov, A.I., Numerically exact quantum dynamics of bosons with time-dependent interactions of harmonic type. *Phys. Rev. A* **86**, 063606 (2012).

[4] Lode, A.U.J., *Tunneling Dynamics in Open Ultracold Bosonic Systems* (Springer, 2014).

[5] Lode, A.U.J., Tsatsos M-C, Fasshauer E, MCTDH-X: The multiconfigurational time-dependent Hartree for indistinguishable particles software, version 0.99. http://ultracold.org; http://mctdhx.org; http://schroedinger.org, http://mctdh.bf (2016).

[6] Beck, M.H., A. Jackle, Worth, G.A. & Meyer, H.-D., The multiconfiguration time-dependent Hartree (MCTDH) method: A highly efficient algorithm for propagating wavepackets. *Phys. Rep.* **34**, 1 (2000).

[7] Doganov,R.A., Klaiman, S., Alon, O.E., Streltsov, A.I. & Cederbaum, L.S., Two trapped particles interacting by a finite-range two-body potential in two spatial dimensions. *Phys. Rev. A* **87**, 033631 (2013).

[8] To convert to dimensionful units, the Hamiltonian is multiplied by $\frac{\hbar^2}{mL^2}$. With the mass $m = 1.44316 \times 10^{-25}$Kg corresponding to Rb^{87}, and length scale $L = 0.750\mu$m, we get a transverse trapping frequency of $\omega_0 = (2\pi)207$Hz and a time scale of 4.84ms. With this choice of scale, $t = 500$ corresponds to $2.42s$, and the linear extent of the simulation is 12μm. The interaction parameter λ_0 is related to the scattering length a_s and transverse confinement $l_z = \sqrt{\frac{\hbar}{m\omega_z}}$ by $\lambda_0 = 2\sqrt{2\pi}\frac{a_s}{l_z}$. For Rb^{87}, $a_s = 90.4a_0$, where a_0 is the Bohr radius. Using this scattering length and $\lambda_0(N-1) = 17.1$ gives $\omega_z = (2\pi)6$kHz for $N = 100$. So the transverse aspect ratio is $\frac{\omega_0}{\omega_z} = 3.43 \times 10^{-2}$ and the maximum energy per particle, $E/\hbar N = 1.41$kHz is merely $3.72 \times 10^{-2}\omega_z$. Thus, we can truly treat the dynamics as two-dimensional.

[9] Sakmann, K., Streltsov, A.I., Alon, O.E. & Cederbaum, L.S., Reduced density matrices and coherence of trapped interacting bosons. *Phys. Rev. A* **78**, 023615 (2008).

[10] Glauber, R.J., The Quantum Theory of Optical Coherence (Wiley-VCH, 2007).

[11] Lundh, E., Martikainen, J.-P. & Suominen, K.-A., Vortex nucleation in Bose-Einstein condensates in time-dependent traps. *Phys. Rev. A* **67**, 063604 (2003).

[12] Dagnino, D., Barberán, N., Lewenstein, M. & Dalibard, J., Vortex nucleation as a case study of symmetry breaking in quantum systems. *Nature Phys.* **5**, 431 (2009).

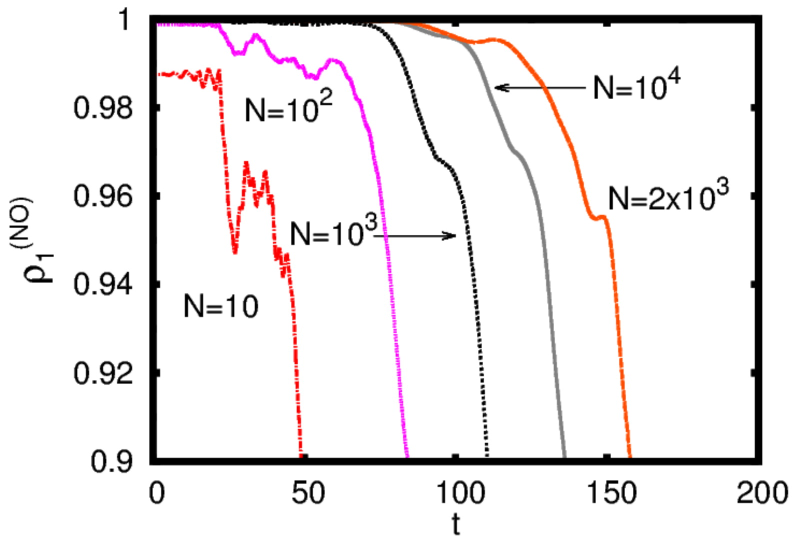

FIG. 1. [COLOR] **Onset of fragmentation at different particle number:** We repeated our calculations varying the particle number, but keeping the mean-field coupling strength $g = \lambda_0(N-1)$ constant with $M = 2$ orbitals. Generally, higher particle numbers fragment at later times. Particle numbers tested up to $N = 10^4$ do fragment during the anisotropy ramp-up or during the period of maximum anisotropy. All quantities shown are dimensionless.

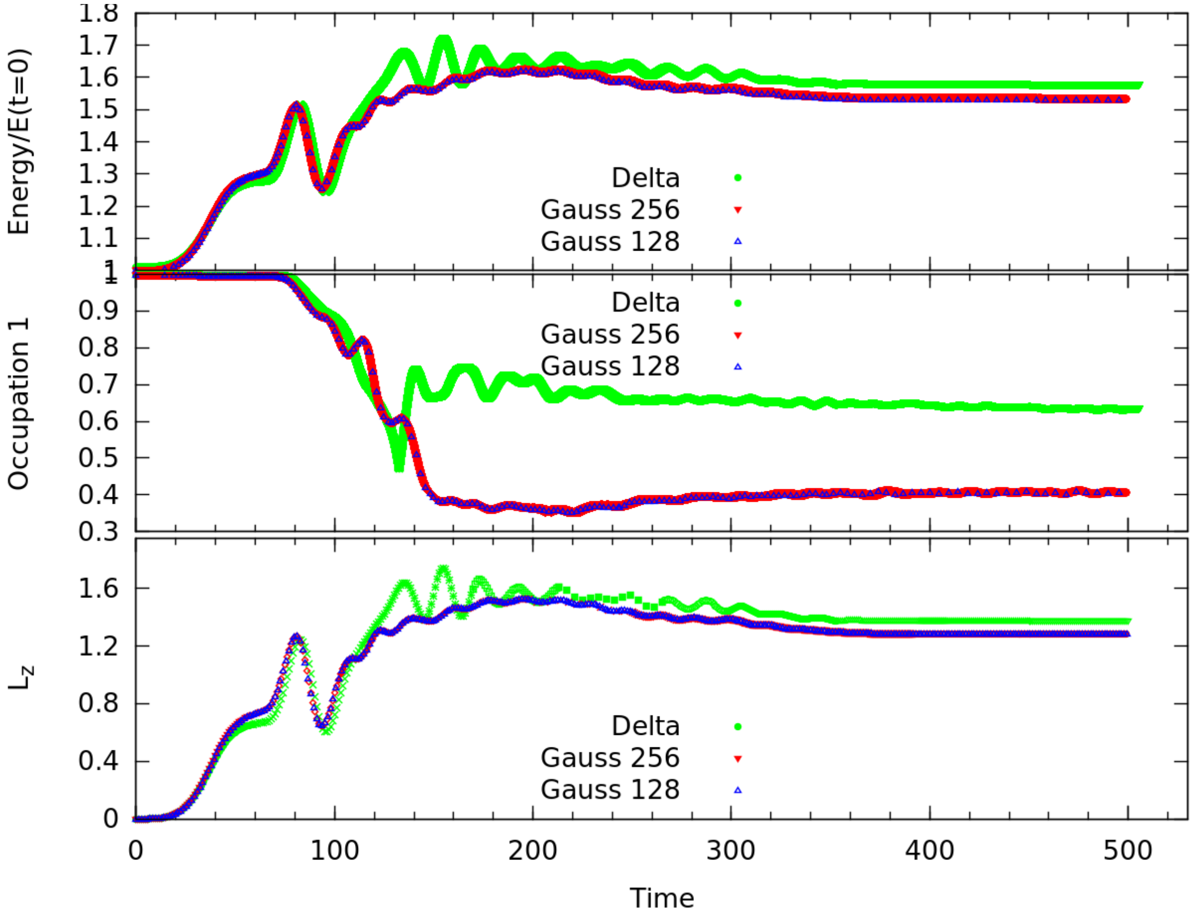

FIG. 2. [COLOR] **Comparisons between a Gaussian interaction model on 128×128 and 256×256 DVR grids and with a delta-pseudopotential interaction model:** The time evolution of system observables and properties (energy, largest natural occupation and angular momentum – upper, middle, and lower panels, respectively) are practically identical for the 128^2 and 256^2 grids. This renders our results exact with respect to the number of grid points. The energy and AM of the system interacting via a delta pseudopotential are very close to its Gaussian counterpart while the largest occupation (at long times) is noticeably larger. All quantities shown are dimensionless.

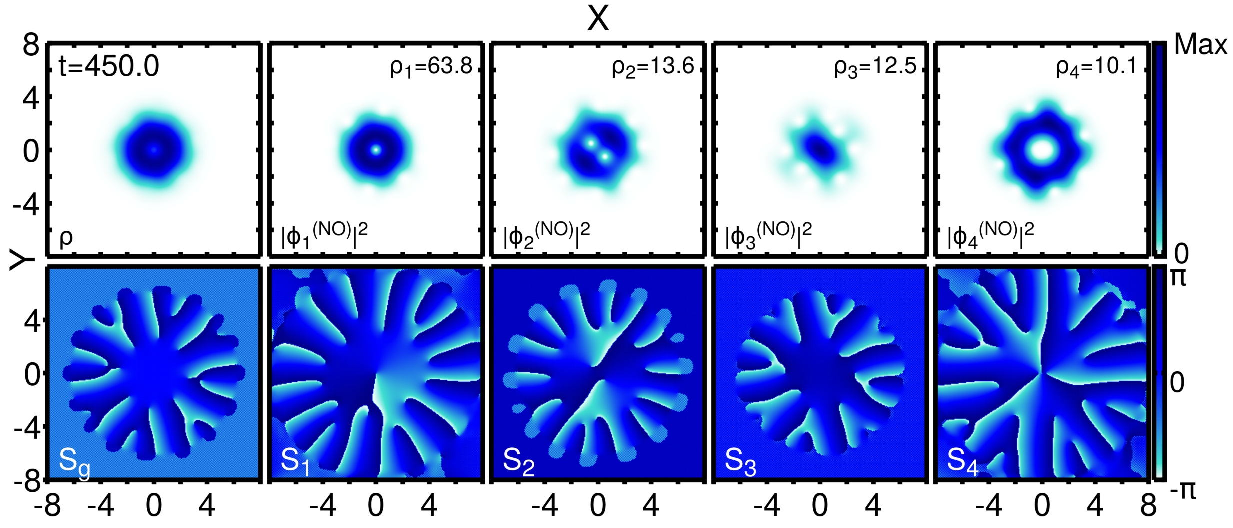

FIG. 3. [COLOR] **Phantom vortices in the system interacting via a contact potential:** Densities at $t = 450.0$ (towards the end of the rotation routine) for the system interacting via a contact pseudopotential (i.e. delta function) (cf. Fig. 3 of the main text – all other parameters besides the interaction type are identical). Qualitatively the system behaves in the same fashion as the one originally presented; the density appears vortex-free (first panel left) while phantom vortices have been nucleated on the first, second and fourth natural orbitals of the same charges as in the case of short-range interactions. Notably, in the case of the contact interaction the first natural occupation is higher than its counterpart in the Gaussian system, resulting in a more pronounced dip in the density in the trap center. All quantities shown are dimensionless.
